# The Association between Olfactory Function and Cognitive Impairment in Older Persons with Cognitive Impairments: A Cross-Sectional Study

**DOI:** 10.3390/healthcare9040399

**Published:** 2021-04-01

**Authors:** Hyangjeong Park, Heejeong Kim, Sisook Kim, Hyegyeong Cha

**Affiliations:** 1 Department of Nursing, Cheonan Medical Center, Cheonan 31071, Chungcheongnam-do, Korea; googooli@hanmail.net; 2 Department of Nursing, College of Health & Health Care, Namseoul University, Cheonan 31020, Chungcheongnam-do, Korea; yshbb@nsu.ac.kr (H.K.); sisookkim@nsu.ac.kr (S.K.)

**Keywords:** cognitive impairment, olfactory function, older person, dementia, CDR, UPSIT

## Abstract

Olfactory function is an emerging topic of research in the fields of cognitive impairment and neurodegenerative diseases. We aimed to confirm the association between olfactory function and cognitive impairment by assessing the olfactory function of older persons with cognitive impairment and identify whether olfactory function is associated with cognitive impairment. For this study, we recruited 117 older people aged ≥65 years with cognitive impairments from a public hospital in Korea. We used the Korean version of the expanded clinical dementia rating scale to evaluate participants’ cognitive impairments, and the University of Pennsylvania’s smell identification test to assess their olfactory function. Our results indicate a significant negative correlation between olfactory function and all domains of cognitive impairment (memory, orientation, judgement and problem-solving, community affairs, home and hobbies, and personal care). In addition, olfactory function was a factor associated with cognitive impairment in older persons. Therefore, we expect that our results to provide useful data for the development of interventions using olfactory stimulation to improve cognitive function in older persons with cognitive impairment.

## 1. Introduction

Since cognitive impairment due to aging progresses slowly, it is difficult to determine the exact timing of onset of dementia. However, once it has progressed to dementia, it causes deterioration in the quality of life for patients and their families, as well as increased medical expenses, leading to high economic burdens at the individual and national levels [1]. However, since there are currently no effective medications or other treatment options for cognitive disorders and dementia, early detection and prompt interventions are important undertakings in the prevention of cognitive impairment. Several previous studies have demonstrated that cognitive impairment in older persons is related to general characteristics such as age, sex, and education [2], lifestyle factors such as drinking and smoking [3], and disease factors such as diabetes [4] and depression [5]. In recent years, many researchers have become increasingly interested in studies on the olfactory impairment observed in the early stages of neurodegenerative diseases, such as Alzheimer’s disease (AD), and Parkinson’s disease (PD), and cognitive impairment [6,7,8,9,10,11].

The human olfactory system includes peripheral sensory neurons in the olfactory epithelium. These send their axons across the cribriform plate of the ethmoids bone to the olfactory bulbs. In the glomerular layer of the olfactory bulbs, these axons synapse with the dendrites of the mitral and tufted cells, which, in turn, project into the main olfactory cortex in the basal forebrain [12]. The olfactory nervous system is composed of complex circuits in which the primary and secondary cortical regions are interconnected and transmitted to the hippocampus, which is responsible for memory, and is also related to attention, conditioning, and spatial perception [13].

Recently, the discovery of neurofibrillary tangles, key pathologic features of Alzheimer’s disease, in the olfactory neurons of the olfactory bulbs in patients with mild AD has resulted in the olfactory function becoming an emerging topic in aging and neurodegenerative diseases research [14,15,16,17]. Furthermore, olfactory deficits in older persons, on their own, may lead to a dislike of food, increased depression, and a lower quality of life [9], and unintentional exposures to toxins such as household gas or fire, which can increase mortality [18]. In addition, even in virus-positive patients such as those infected with the SARS-CoV-2 virus, olfactory impairment has appeared as an early symptom [9]. Accordingly, there is now an increasing interest in olfactory function testing.

The olfactory function tests include an identification test (to identify the odour), a threshold test (to determine the lowest concentration of odour that can be perceived), and a discrimination test (to distinguish between different odours) [19]. According to several reports, while the olfactory threshold is generally maintained in the early stages of cognitive impairment, the olfactory identification ability is reduced [10,11,16,20,21]. Increasing evidence [9,11,16,22,23] demonstrates that olfactory identification deficiencies are associated with cognitive impairment, including the transition from normal cognition to mild cognitive impairment (MCI). However, a meta-analysis [24] revealed that the association between olfactory function and neurodegenerative diseases was small.

Therefore, we conducted this study to determine whether the olfactory function is related to the cognitive impairment of older persons by analysing the relationship between the olfactory identification test and the detailed areas of cognitive function in older persons with cognitive impairment. This study can provide useful data to determine whether olfactory stimulation can be used as an intervention method for improving cognitive function of older persons in the future.

## 2. Methods

### 2.1. Participants

We conducted this study from April to September 2019 according to the approved guidelines and screening procedures of Namseoul University, Cheonan (IRB No: 1041479-HR-20190-002). We recruited participants aged ≥65 years from the Cheonan Medical Center in Korea who were suspected of having cognitive impairment. The inclusion criteria were limited to patients who were capable of communication and who could perform olfactory and cognitive function tests. The exclusion criteria were as follows: (1) Patients with olfactory and taste-related diseases that could affect olfactory function test, such as active rhinitis, chronic sinusitis, and intracranial polyps; (2) Patients with diseases that could affect the assessment of olfactory and cognitive functions due to cerebral neuropsychological causes, such as severe depression, schizophrenia, and a history of brain damage; and (3) patients who took medications that could affect olfactory and palate functions, such as antirheumatic or anticancer medications.

The sample size was determined using G* power software (G* Power 3.1.9.2, Heinrich-Heine-University, Düusseldorf, Germany), which indicated that, for correlation analysis with an effect size of 0.3, a significance level (α) of 0.05, and power (1 − β) of 0.95, the required sample size was 115. Thus, 117 participants (75.72 ± 6.63, 58 men, 59 women) recruited.

### 2.2. Research Variables

The general characteristics of the participants included sex, age, marital status, education period, religion, cohabitation type, and smoking and alcohol consumption.

#### 2.2.1. Expanded Clinical Dementia Rating

The Korean version of the expanded clinical dementia rating (CDR) [25] was used to distinguish and evaluate participants’ comprehensive cognitive levels. The expanded CDR consists of a five-point Likert scale, which evaluates six functions in detail: (1) memory, (2) orientation, (3) judgment and problem-solving, (4) community affairs, (5) home and hobbies, and (6) personal care. First, through detailed interviews with participants and guardians, the examiner identified the functions of the six areas and then determined the scores for each area. Points from 0 to 5 (0, 0.5, 1, 2, 3, 4, and 5) were allocated for each of the six areas. A CDR score of 0 indicated “none”; 0.5, “questionable”; 1, “mild”; 2, “moderate”; 3, “severe”; 4, “profound”; and 5, “terminal” dementia. Second, the sum of boxes (CDR-SB) was calculated, which is the sum of the scores in all six areas. The higher the score, the more severe the cognitive impairment. In this study, the expanded CDR was evaluated by a trained nurse and finally deciphered by a neurologist. The Kappa value, which indicates reliability among examiners, ranged from 0.90 to 0.96, showing a high degree of agreement at the level of “almost perfect” (1.0 ≥ k ≥ 0.8).

#### 2.2.2. Olfactory Identification Test

The University of Pennsylvania smell identification test (UPSIT) was used as the olfactory identification test [26]. This test was conducted using 40 “scratch and sniff” odorants. The odorants were embedded in microcapsules and positioned on brown strips at the bottom of the test booklets. The stimuli were released by scratching the strips with a pencil tip in a standardized manner. Each odorant strip contained a multiple-choice question with four different responses. The participants were required to mark one of the four responses. The test duration was approximately 10–15 min, and the score was calculated as the sum of the correct scores for 40 odours. The diagnostic criteria of UPSIT were as follows [27]: a score of 0–18 indicates, “total anosmia”; 19–25, “severe microsmia”; 26–29 (males) or 26–30 (females), “moderate microsmia”; 30–33 points (males) or 31–34 (females), “mild microsmia”; and 34–40 (males) or 35–40 (females), “normosmia.” In this classification scheme, “anosmia” was defined as a total inability to perceive a qualitative odour stimulus. The term “microsmia” describes the condition of lessened smell function in the typical clinical situation and is subdivided into “severe,” “moderate,” and “mild. “Normosmia” indicates normal olfactory sense. At the time of development, a test–retest reliability (r) value of 0.95 was calculated using the Spearman–Brown formula. The higher the score, the better the olfactory function.

### 2.3. Statistical Analysis

The collected data were analysed using SPSS 21.0 for Windows (IBM Corp, Armonk, NY, USA). The participants’ general characteristics, olfactory functions, and cognitive impairments were analysed using descriptive statistics, including means, standard deviations, frequencies, and percentages. The *t*-test, analysis of variance (ANOVA), and Welch test were used to analyse the differences in olfactory functions and cognitive impairments according to the general characteristics, and the Scheffé test and Games–Howell test were used for the post hoc analysis. The relationship between participants’ olfactory functions and cognitive impairments was analysed using the Pearson correlation coefficient. Hierarchical regression analysis was performed to identify factors associated with cognitive impairment. The significance level for the statistical tests was set at 0.05.

## 3. Results

### 3.1. Differences in Olfactory Function and Cognitive Impairment According to Participants’ General Characteristics

We identified differences in olfactory functions and cognitive impairments among participants according to sex, age, marital status, education period, religion, cohabitation type, smoking, and alcohol consumption. There were significant differences in olfactory function according to age, education period, and cohabitation type. We also observed significant differences in cognitive impairment according to sex, age, education period, and cohabitation type (Table 1).

### 3.2. The Degree of Participants’ Olfactory Function and Cognitive Impairment

Table 2 shows the degree of participants’ olfactory functions and cognitive impairments. In total, 58 (49.6%) participants had “total anosmia”, 40 (34.2%) had “severe anosmia”, 16 (13.7%) had “moderate microsmia”, and three (2.6%) had “mild microsmia”. None of the participants had “normosmia”. Regarding participants’ cognitive impairment, 69 (59.0%) had “questionable dementia”, 36 (30.8%) had “mild dementia”, and 12 (10.0%) had “moderate dementia”. None of the participants had “severe dementia”, “profound dementia”, or “terminal dementia”.

### 3.3. Correlation between Olfactory Function and Cognitive Impairment

Table 3 shows the correlations between the olfactory functions and cognitive impairments of the participants. There was a significant negative correlation between the olfactory function and all the domains of cognitive impairment: memory, orientation, judgement and problem-solving, community affairs, home and hobbies, and personal care. The results indicate that the worse the olfactory function, the more severe the cognitive impairment.

### 3.4. Factors Associated with Cognitive Impairment in Older Persons with Cognitive Impairment

The aptness of the regression equation was confirmed to be acceptable with a tolerance of 0.765–0.929, a variance inflation factor (VIF) of 1.077–1.308, and a Durbin–Watson statistic of 1.994. To confirm whether the olfactory function was a factor associated with cognitive impairment when controlling sex, age, education period, and cohabitation type, which demonstrated significant differences in Table 1, we performed a hierarchical regression analysis (Table 4). First, we inputted sex, age, education period, and cohabitation type as independent variables and cognitive impairment as a dependent variable in model 1. The results indicated that age (β = 0.509, *p* < 0.001) and cohabitation type (spouse and their families) (β = 0.193, *p* = 0.024) were significant, and the explanatory power of these two variables on cognitive impairment was 36.6%. We then added olfactory function as an independent variable in model 2. The results indicated that age (β = 0.396, *p* < 0.001) and olfactory function (β = −0.348, *p* < 0.001) were significant, and the explanatory power increased to 45.3%. Therefore, we confirmed that olfactory function is a factor associated with cognitive impairment in older persons with cognitive impairment.

## 4. Discussion

This study aimed to confirm the association between olfactory function and cognitive impairment in older persons and whether olfactory function is associated with cognitive impairment. We analysed the differences in olfactory function and cognitive impairment according to general characteristics, the correlation between olfactory function and cognitive impairment, and factors associated with cognitive impairment.

Olfactory function in older persons was observed to vary according to age, education period, and cohabitation type; cognitive impairment also differed according to sex, age, education period, and cohabitation type. Age is generally the most important variable affecting olfactory and cognitive function [6]. Many recent studies have demonstrated that cognitive impairment in older persons is accompanied by a decrease in olfactory function [7,8,9,10,11]. Therefore, in this study, it is considered a natural result that reduced olfactory function and cognitive impairment were more common among participants over 75 years of age than those who were younger. As for the difference in cognitive impairment according to sex, similar to a previous study [3], confirmed that the proportion of women with cognitive impairment was higher than for men in this study. However, unlike previous studies in which women were displayed superior olfactory function [26,28], our results did not indicate a significant sex difference in olfactory function. One study [29] reported that the number of cells in olfactory regions is far higher among women than men. However, since the olfactory function is highly influenced by the environment, we suspect that the difference is due to the environment’s effect. Therefore, there is a need to accumulate data through repeated studies. Olfactory function and cognitive impairment differed according to cohabitation type. The olfactory function score was lower and the cognitive impairment was higher for those living with a spouse and their family members than those living with only the spouse. However, these results are not interpreted as being influenced by family members living together, but rather we suppose that older persons who experience daily life difficulties due to severe cognitive impairment often reside together with their families for caregiving purposes.

An important result of this study is the relationship between olfactory function and cognitive impairment. Although several previous studies report a relationship between olfactory function and cognitive impairment [7,8,9,10,11,21,30], to the best of our knowledge there are no studies that confirm the relationship between olfactory function and detailed areas of cognitive function. Therefore, we identified the relationship between olfactory function and cognitive impairment based on the UPSIT tool, which assesses olfactory identification function, and the expanded CDR, which is a tool for distinguishing overall cognitive function level. Our results indicate that olfactory function was related to all areas of the orientation, judgment and problem-solving, community affairs, home and hobbies, personal care, and memory domains of the CDR. Furthermore, we analysed whether olfactory function is associated with cognitive impairment after controlling for sex, age, education period, and cohabitation type, which are the general characteristics that displayed differences with respect to cognitive impairment, and the results were significant. However, although this study confirmed that olfactory function is one factor associated with cognitive impairment, the possibility that the olfactory test was affected by the participant’s cognitive impairment cannot be excluded.

Recently, it has been demonstrated that olfactory stimulation helps improve memory, recall of past events, and planning of future events [31,32,33]. Further, the current findings revealed that olfactory function is related to various sub-areas of cognitive function. Therefore, we believe our findings provide useful data to develop an intervention using olfactory stimulation to improve memory, orientation, judgment and problem-solving, community affairs, home and hobbies, and personal care functions of older persons with cognitive impairment in the future.

There are several limitations to this study. First, the results of this study should be generalized with caution, as our participants included only older persons with cognitive impairment who visited a single hospital in Korea. Second, in order to participate in this study, participants were required to be capable of communication; therefore, people with severe cognitive impairment were not eligible for participation. Third, we used the UPSIT for the olfactory identification test in this study. However, participants had difficulty distinguishing odours because of unfamiliar odorants and some participants had difficulty choosing even one from the four odorants due to lower education levels. Given that we were unable to locate an olfactory identification test tool developed specifically for older persons, we used the UPSIT tool, as it is most widely used. Therefore, to accurately test the olfactory function of older persons with cognitive impairment, it is necessary to develop an olfactory test tool that considers cognitive impairment, lower education level, and odorants familiar to older people.

## 5. Conclusions

Despite these limitations, we were able to confirm the relationship between impairment of memory, orientation, judgment and problem solving, community affairs, home and hobbies, and personal care, which are the subdomains of cognitive function, and olfactory function among older persons with cognitive impairment. In addition, we confirmed that olfactory function is a factor associated with cognitive impairment. We believe that our results can provide useful data for the development of interventions using olfactory stimulation to improve cognitive function in older people with cognitive impairment. Repeated studies are necessary to extend the generalizability of the results of this study, and the development of an olfactory test tool for older people with cognitive impairment is also necessary.

## Figures and Tables

**Table 1:** Differences in olfactory function and cognitive impairment according to general characteristics.

Characteristics	Categories	N (%), Mean ± SD	Olfactory Function (*n* = 117)		Cognitive Impairment (*n* = 117)	
			Mean ± SD	t or F (*p*-Value) Scheffé	Mean ± SD	t or F (*p*-Value) Scheffé/Games–Howell
Sex	Men	58 (49.6)	18.09 ± 7.42	1.53 (0.128)	4.10 ± 2.16	5.02 (0.027) ^†^
	Women	59 (50.4)	15.78 ± 8.77		5.22 ± 3.16	
Age (year)	Average	75.72 ± 6.63				
	<75	54 (46.2)	19.17 ± 7.03	2.73 (0.006) *	3.58 ± 1.96	19.28 (<0.001) ^†^
	≥75	63 (53.8)	15.00 ± 8.64		5.61 ± 3.00	
Marital status	Married	68 (58.1)	18.00 ± 8.26	1.69 (0.093)	4.32 ± 2.51	2.69(0.104)
	Unmarried	49 (41.9)	15.43 ± 7.91		5.16 ± 3.02	
Education period (year)	0–3 ^a^	27 (23.1)	12.56 ± 9.47	4.77 (0.001) * e > a	6.21 ± 3.63	4.02 (0.009) * a > d, e
	4–6 ^b^	47 (40.2)	16.30 ± 6.91		4.38 ± 1.97	
	7–9 ^c^	17 (14.5)	19.24 ± 7.73		4.85 ± 3.45	
	9–12 ^d^	17 (14.5)	20.00 ± 7.14		3.88 ± 1.38	
	≥12 ^e^	9 (7.7)	23.11 ± 6.39		2.67 ± 1.77	
Religion	Yes	55 (47.0)	16.84 ± 8.22	0.108 (0.915)	4.69 ± 2.90	0.01 (0.934)
	None	62 (53.0)	17.00 ± 8.21		4.65 ± 2.65	
Cohabitation type	None ^a^	37 (31.6)	16.59 ± 7.40	5.34 (0.006) * b > c	4.44 ± 2.41	4.03 (0.024) ^†^ c > b
	Only spouse ^b^	57 (48.7)	18.91 ± 8.14		4.09 ± 2.17	
	Spouse and their families ^c^	23 (19.7)	12.52 ± 7.98		6.48 ± 3.77	
Smoking	None	106 (90.6)	17.26 ± 8.23	1.11 (0.334)	4.68 ± 2.76	0.26 (0.775)
	<Half a pack	7 (6.0)	14.57 ± 7.00		4.14 ± 1.35	
	<A pack	4 (3.4)	12.00 ± 8.29		5.38 ± 4.68	
Alcohol consumption	None	91 (77.8)	16.21 ± 8.36	0.61 (0.659)	4.95 ± 2.86	1.06 (0.381)
	Several times a year	3 (2.6)	19.67 ± 7.09		4.50 ± 0.50	
	Several times a month	4 (3.4)	18.75 ± 4.99		3.00 ± 1.29	
	Several times a week	14 (12.0)	19.07 ± 8.52		3.86 ± 2.61	
	Every day	4 (3.4)	19.00 ± 4.69		3.63 ± 1.70	

^†^ Welch test; * *p* < 0.05. SD, standard deviation; ^a, b, c, d, e^ indicate letters for Post-hoc test of ANOVA.

**Table 2:** Participants’ olfactory function and cognitive impairment.

Variables	Scales	Categories	Score Range or Score Level	N (%) or Mean ± SD
Olfactory function (*n* = 117)	UPSIT	Total anosmia	0–18	58 (49.6)
		Severe anosmia	19–25	40 (34.2)
		Moderate microsmia	26–29	16 (13.7)
		Mild microsmia	30–33	3 (2.6)
		Normosmia	34–40	0 (0.0)
Cognitive impairment (*n* = 117)	CDR-SB		0–30	4.67 ± 2.76
	CDR	None	0	0 (0.0)
		Questionable	0.5	69 (59.0)
		Mild	1	36 (30.8)
		Moderate	2	12 (10.0)
		Severe	3	0 (0.0)
		Profound	4	0 (0.0)
		Terminal	5	0 (0.0)

SD, standard deviation; UPSIT, University of Pennsylvania smell identification test; CDR-SB, clinical dementia rating–sum of boxes; CDR, clinical dementia rating.

**Table 3:** Correlation between olfactory function and cognitive impairment.

Variables		UPSIT	CDR Sub-Scale					
			1	2	3	4	5	6
UPSIT		1						
CDR sub-scale	1	−0.473 **	1					
	2	−0.477 **	0.583 **	1				
	3	−0.526 **	0.727 **	0.585 **	1			
	4	−0.566 **	0.744 **	0.566 **	0.732 **	1		
	5	−0.541 **	0.668 **	0.467 **	0.682 **	0.766 **	1	
	6	−0.442 **	0.672 **	0.422 **	0.604 **	0.714 **	0.769 **	1

** *p* < 0.01. UPSIT, University of Pennsylvania smell identification test; CDR, clinical dementia rating. CDR sub-scale: 1: memory, 2: orientation, 3: judgement and problem-solving, 4: community affairs, 5: home and hobbies, 6: personal care.

**Table 4:** Factors associated with cognitive impairment in older persons with cognitive impairment.

Variable	Model 1					Model 2				
	B	S.E.	β	t	*p*-Value	B	S.E.	β	t	*p*-Value
(Constant)	−11.512	2.788		−4.129	<0.001	−6.357	2.852		−2.229	0.028
Sex	0.633	0.495	0.115	1.279	0.203	0.737	0.460	0.134	1.601	0.112
Age	0.212	0.033	0.509	6.334	<0.001 **	0.164	0.033	0.396	4.998	<0.001 **
Education period	−0.109	0.209	−0.048	−0.523	0.602	0.079	0.199	0.035	0.399	0.691
Cohabitation (only spouses) ^†^	−0.512	0.519	−0.087	−0.987	0.326	−0.562	0.482	−0.095	−1.166	0.246
Cohabitation (spouse and their families) ^†^	1.331	0.581	0.193	2.290	0.024 *	0.873	0.550	0.126	1.585	0.116
Olfactory function						−0.117	0.027	−0.348	−4.317	<0.001 **
	F (*p*) = 14.398 ** R2 = 0.393, Adjusted R2 = 0.366, ΔR2 = 0.393					F (*p*) = 17.010 ** R2 = 0.481, Adjusted R2 = 0.453, ΔR2 = 0.088				

^†^ Dummy variable; * *p* < 0.05; ** *p* < 0.001. S.E., standard error; Model 1 included general characteristics as independent variables; Model 2 included general characteristics and olfactory function as independent variables.

## Data Availability

The data that support the findings of this study are available on request from the corresponding author, H.C. The data are available on request due to ethical restrictions.
